# Systemic, Pleural, and Alveolar Cytokine Responses After Video-Assisted Thoracoscopic Versus Open Lobectomy for Non-Small Cell Lung Cancer: A Prospective Observational Study

**DOI:** 10.3390/jcm15197579

**Published:** 2026-09-29

**Authors:** Burcu Ancın, Mesut Melih Özercan, Yiğit Yılmaz, Serkan Uysal, Ulaş Kumbasar, Erkan Dikmen, Zeynep Sarıbaş, Rıza Doğan

**Affiliations:** 1 Department of Thoracic Surgery, Faculty of Medicine, Hacettepe University, Ankara 06100, Türkiye; 2 Department of Thoracic Surgery, Faculty of Medicine, Kırıkkale University, Kırıkkale 71450, Türkiye; 3 Department of Medical Microbiology, Faculty of Medicine, Hacettepe University, Ankara 06100, Türkiye

**Keywords:** non-small cell lung cancer, cytokines, lobectomy, open thoracotomy, video-assisted thoracoscopic surgery, one-lung ventilation

## Abstract

**Background/Objectives**: Surgical access and one-lung ventilation (OLV) may generate distinct systemic and local inflammatory responses after lung resection. We compared perioperative cytokine profiles in serum, pleural fluid, and bronchoalveolar lavage (BAL) after video-assisted thoracoscopic surgery (VATS) and open thoracotomy for non-small cell lung cancer (NSCLC). **Methods**: This prospective, single-center, non-randomized observational study included 40 adults undergoing anatomical lobectomy with mediastinal lymph-node dissection (VATS, *n* = 20; open thoracotomy, *n* = 20). Surgical access was selected according to patient preference. TNF-α, IL-6, IL-8, and IL-10 were measured in serial serum and pleural-fluid samples and in BAL from both lungs before OLV and after resection. **Results**: Serum and pleural-fluid cytokines peaked at 4 h in both groups. Exploratory pointwise comparisons showed that at 24 h, serum TNF-α (4.89 [IQR, 4.43–5.92] vs. 4.01 [IQR, 3.75–5.12] pg/mL; *p* = 0.010), IL-6 (150.22 [IQR, 136.90–194.66] vs. 108.00 [IQR, 100.46–125.88] pg/mL; *p* < 0.001), and IL-8 (66.83 [IQR, 57.70–82.82] vs. 43.62 [IQR, 32.89–50.59] pg/mL; *p* = 0.001) were higher after thoracotomy than after VATS; differences at 48 h persisted for TNF-α (2.48 [IQR, 1.86–3.04] vs. 1.99 [IQR, 1.19–2.23] pg/mL; *p* = 0.010) and IL-8 (16.92 [IQR, 15.09–23.99] vs. 7.42 [IQR, 6.44–11.30] pg/mL; *p* < 0.001). Pleural-fluid IL-6 at 24 h and IL-8 at 48 h were also higher after thoracotomy, whereas BAL cytokines did not differ significantly by approach. Longitudinal GEE analyses showed significant approach-by-time interactions for serum TNF-α, IL-6, and IL-8 and for pleural-fluid IL-8, but not for pleural-fluid IL-6. **Conclusions**: VATS was associated with a lower systemic proinflammatory response than open thoracotomy, while differences in the pleural compartment were more limited and no detectable difference was observed in early alveolar cytokine responses. These findings highlight compartment-specific differences in perioperative inflammatory responses between VATS and open thoracotomy.

## 1. Introduction

Surgical trauma induces a complex inflammatory response resulting from the interplay between tissue injury, perioperative stress, and immune activation, with subsequent effects on postoperative morbidity and clinical outcomes [1]. In lung resections, this response differs from other surgical procedures because of direct manipulation of the lung parenchyma, opening of the pleural cavity, and one-lung ventilation [2,3]. Therefore, the difference in tissue trauma between minimally invasive approaches and open surgery is considered one of the key factors influencing perioperative inflammation [4].

In surgically eligible patients with early-stage non-small cell lung cancer (NSCLC), curative treatment is primarily based on anatomical lung resection [5,6]. Video-assisted thoracoscopic surgery (VATS) has increasingly been adopted in lung cancer surgery as a minimally invasive approach that aims to limit surgical trauma and perioperative morbidity without compromising oncological principles [7]. However, inflammatory differences between VATS and open surgery, particularly when systemic and local compartments are assessed together, remain incompletely clarified.

Tumor necrosis factor-alpha (TNF-α), interleukin-6 (IL-6), interleukin-8 (IL-8), and interleukin-10 (IL-10) are key biochemical markers reflecting different aspects of inflammation in thoracic surgery and lung injury. IL-6 is associated with the acute-phase response, surgical trauma severity, and postoperative complications [8,9]. IL-8 contributes to pulmonary inflammation and alveolar injury through neutrophil chemotaxis and activation [4,10]. TNF-α is a major mediator of the early proinflammatory response, whereas IL-10 regulates this process through anti-inflammatory effects [11,12].

Although VATS has been associated with a lower postoperative cytokine response than open thoracotomy, circulating cytokine measurements alone cannot determine whether this attenuation also extends to the local pleural and alveolar environments [4]. Pleural fluid reflects inflammatory activity adjacent to the operative field and may therefore be influenced by pleural manipulation, chest-wall trauma, and pulmonary resection. In contrast, bronchoalveolar lavage reflects lung-specific inflammatory processes associated with one-lung ventilation, including mechanical stress in the ventilated lung and hypoxia, surgical manipulation, and re-expansion in the collapsed lung [2,13].

Systemic, pleural, and alveolar inflammatory responses may not change in parallel, and relatively few studies have evaluated these biological compartments concurrently [2,13]. The contribution of this study lies in combining serial serum and pleural-fluid measurements during the first 48 postoperative hours with bilateral BAL assessment, using the same cytokine panel in a prospective lobectomy cohort. Accordingly, the primary objective was to compare the perioperative systemic inflammatory response between VATS and open thoracotomy, while the secondary objective was to determine whether differences related to surgical approach were also present in the local pleural and alveolar compartments.

## 2. Materials and Methods

### 2.1. Study Design and Patient Selection

This prospective, single-center, non-randomized observational study was conducted at the Department of Thoracic Surgery, Hacettepe University Faculty of Medicine, between January 2019 and December 2021. Patients who met the eligibility criteria described below and were considered technically suitable for either VATS or open lobectomy received standardized information regarding both surgical approaches. The surgical approach was selected according to patient preference after discussion with the surgical team. Consecutive eligible patients who selected either VATS or open thoracotomy and provided written informed consent were enrolled in the corresponding surgical cohort until the planned target of 20 patients per group was reached. No random allocation or matching was performed. All enrolled patients underwent anatomical lobectomy with systematic mediastinal lymph node dissection. Patients aged ≥18 years with suspected or histopathologically confirmed primary NSCLC, clinically resectable stage I or II disease based on preoperative thoracic computed tomography, 18F-fluorodeoxyglucose (18F-FDG) positron emission tomography/computed tomography, and brain magnetic resonance imaging, and scheduled for elective anatomical lobectomy were eligible for inclusion. Final inclusion required histopathological confirmation of primary NSCLC, established either by preoperative transthoracic fine-needle aspiration or core needle biopsy, or by intraoperative frozen-section examination. Pathological nodal status was determined by examination of the systematic lymph node dissection specimens. Exclusion criteria were secondary pulmonary malignancy or distant metastatic disease; receipt of neoadjuvant chemotherapy or radiotherapy; active systemic or respiratory tract infection, including pneumonia; acute exacerbation of chronic obstructive pulmonary disease or asthma at the time of enrollment; known chronic inflammatory, autoimmune, or rheumatologic disease; immunodeficiency; and chronic systemic corticosteroid or other immunosuppressive treatment. This study is reported in accordance with the Strengthening the Reporting of Observational Studies in Epidemiology (STROBE) statement.

The study protocol was approved by the Hacettepe University Ethics Committee (Project No: GO 19/261; Decision No: 2019/09-03). The study was conducted in accordance with the principles of the Declaration of Helsinki, as revised in 2013. All patients were informed in detail about the surgical treatment and study-related sampling procedures, and written informed consent was obtained from each participant before inclusion in the study.

### 2.2. Anesthesia, Surgical Technique, and Postoperative Analgesia

All patients underwent general anesthesia with one-lung ventilation and were placed in the lateral decubitus position. Intraoperative ventilation was managed according to standardized lung-protective principles. Respiratory rate, tidal volume, positive end-expiratory pressure, and fraction of inspired oxygen were adjusted to maintain adequate oxygenation and normocapnia. Intraoperative monitoring included electrocardiography, pulse oximetry, continuous invasive arterial blood pressure monitoring, and arterial blood gas analysis.

All operations were performed by an experienced thoracic surgery team according to the same oncological principles. Open lobectomy was performed through a standard posterolateral thoracotomy, whereas VATS lobectomy was conducted using a uniportal thoracoscopic approach without rib spreading. In both groups, anatomical lobectomy involved individual dissection and division of the lobar pulmonary vessels and bronchus. Systematic mediastinal lymph node dissection was performed according to standard oncological practice in all patients.

After completion of the resection, hemostasis and air leakage were assessed, the remaining lung was re-expanded under direct visualization, and a single chest tube was placed. Postoperative analgesia was administered according to the same institutional multimodal analgesia protocol in both groups and consisted of systemic non-steroidal anti-inflammatory drugs and opioid analgesics. This standardized approach was used to minimize the potential confounding effects of postoperative pain and analgesic exposure on inflammatory mediator levels.

### 2.3. Clinical Data Collection

Demographic, clinical, surgical, and pathological data were recorded using a standardized form. Age, sex, smoking history, comorbidities, resected lobe, operation time, chest tube removal time, surgical approach, histopathological subtype, clinical and pathological stage, and pathological nodal status were evaluated.

### 2.4. Collection of Peripheral Blood, Pleural Fluid, and Bronchoalveolar Lavage Samples

Peripheral blood, pleural fluid, and BAL samples were collected using a standardized protocol. Peripheral venous blood was obtained preoperatively, before induction of general anesthesia, and at postoperative 4, 24, and 48 h. Pleural fluid was obtained before surgical manipulation and at postoperative 4, 24, and 48 h. After thoracic access was achieved and before surgical manipulation, the baseline pleural sample was obtained following pleural lavage with 100 mL of sterile saline; postoperative samples were collected from fluid accumulated in the chest tube line after brief chest tube clamping.

Bronchoalveolar lavage specimens were obtained at two intraoperative time points by the same investigator using a fiberoptic bronchoscope introduced through the double-lumen endotracheal tube. Baseline samples were collected after induction of general anesthesia and intubation, but before surgical incision and initiation of one-lung ventilation. For sample classification, the dependent contralateral lung that would remain ventilated during one-lung ventilation was designated as the ventilated lung, whereas the operative lung that would subsequently be collapsed was designated as the collapsed lung. The bronchoscope was advanced sequentially into the corresponding main bronchus of each lung. A 20-mL aliquot of sterile 0.9% saline was instilled through the working channel of the bronchoscope and immediately re-aspirated into separate sterile collection containers for each lung.

The second bronchoscopic sampling was performed after completion of lobectomy and systematic mediastinal lymph node dissection and re-expansion of the operative lung. The patient remained under general anesthesia and intubated with the double-lumen endotracheal tube throughout the sampling procedure, which was undertaken immediately before extubation. The bronchoscope was advanced sequentially into the main bronchus of the previously ventilated non-operative lung and the main bronchus of the previously collapsed operative lung. For each lung, a fresh 20-mL aliquot of sterile 0.9% saline was instilled and immediately re-aspirated into a separate sterile collection container. Each specimen was labeled according to the side and the ventilation status of the lung during one-lung ventilation. Thus, in the post-resection samples, the terms “ventilated lung” and “collapsed lung” refer to the ventilation status of the respective lungs during the preceding period of one-lung ventilation rather than their condition at the time of sampling.

All blood, pleural fluid, and lavage samples were centrifuged at 2500 rpm for 15 min. Serum and supernatant fractions were stored at −80 °C until cytokine analysis. Sampling and preanalytical procedures were performed by the same investigator.

### 2.5. Measurement of TNF-α, IL-6, IL-8, and IL-10 Levels by ELISA

Cytokine concentrations, including TNF-α, IL-6, IL-8, and IL-10, were quantified in peripheral blood, pleural fluid, and BAL specimens by enzyme-linked immunosorbent assay (ELISA), following the manufacturers’ instructions. TNF-α, IL-6, and IL-10 were measured using ELISA kits from DIAsource ImmunoAssays SA, Louvain-la-Neuve, Belgium, with manufacturer-reported analytical sensitivities of 0.7, 2.0, and 1.6 pg/mL, respectively. IL-8 was measured using an ELISA kit from Cloud-Clone Corp., Katy, TX, USA, with an analytical sensitivity of 5.9 pg/mL. These analytical sensitivities were used as the assay-specific limits of detection (LODs). Values reported as “min,” recorded as 0.00, or below the assay-specific LOD were replaced with one-half of the corresponding detection limit (LOD/2) for statistical analysis: 0.35 pg/mL for TNF-α, 1.00 pg/mL for IL-6, 2.95 pg/mL for IL-8, and 0.80 pg/mL for IL-10. Values at or above the LOD were analyzed as measured. Cytokine concentrations were calculated from standard curves, and all measurements were performed in duplicate.

### 2.6. Study Outcomes

The primary outcome was the between-group difference in the systemic inflammatory response, assessed by serum TNF-α, IL-6, IL-8, and IL-10 levels at the predefined perioperative sampling time points. Changes in serum cytokine levels over time within each surgical group were also evaluated.

The secondary outcomes were the between-group differences in pleural and alveolar inflammatory responses at the predefined sampling time points. The pleural inflammatory response was assessed using pleural fluid cytokine levels, whereas the alveolar inflammatory response was evaluated using cytokine levels in bronchoalveolar lavage samples obtained separately from the ventilated and collapsed lungs.

### 2.7. Statistical Analysis

Data were analyzed using IBM SPSS Statistics version 22.0 (IBM Corp., Armonk, NY, USA), with statistical significance set at a two-sided *p*-value < 0.05. Normality of continuous variables was assessed using histograms, skewness and kurtosis values, and the Shapiro–Wilk test. Normally distributed continuous variables were summarized as mean ± standard deviation and compared using the independent-samples *t*-test, whereas non-normally distributed variables were summarized as median (interquartile range [IQR]) and compared using the Mann–Whitney U test. Cytokine concentrations showed non-normal distributions; therefore, descriptive and pointwise comparisons were based on nonparametric methods. All statistical analyses of cytokine concentrations were performed after applying the prespecified LOD/2 substitution procedure to values below the assay-specific LOD.

Categorical variables were compared using the Pearson chi-square or Fisher’s exact test, as appropriate. Cytokine levels were analyzed separately by compartment and sampling time. Within-group temporal changes were assessed using the Friedman test, whereas between-group comparisons at each prespecified time point were performed using the Mann–Whitney U test and were considered exploratory. Longitudinal changes in serum and pleural-fluid cytokines were additionally evaluated using generalized estimating equation (GEE) models to assess whether cytokine trajectories differed between surgical approaches. Surgical approach, sampling time, and the approach-by-time interaction were included as model effects, with time treated as a categorical variable and patient as the clustering variable. An autoregressive [AR(1)] working correlation structure was used to account for within-patient repeated measurements. The approach-by-time interaction was used to assess whether cytokine trajectories differed between the surgical groups. As a sensitivity analysis for potential residual confounding, the longitudinal GEE models were additionally adjusted for age, smoking history (ever vs. never), cardiac comorbidity, and pulmonary comorbidity. No adjustment for multiple testing was applied across cytokines, compartments, or sampling time points; therefore, *p*-values from the cytokine analyses were considered nominal, and the findings were interpreted as exploratory. The target sample size of 20 patients per group was not derived from a formal a priori power calculation. For this prespecified sample size, a sensitivity analysis indicated approximately 80% power to detect a large between-group effect (d ≈ 0.94) at a two-sided alpha level of 0.05.

## 3. Results

### 3.1. Patient Characteristics

A total of 40 eligible and consenting patients were prospectively enrolled, including 20 patients in the open thoracotomy cohort and 20 patients in the VATS cohort. Enrollment was completed when the planned target of 20 patients had been reached in each surgical cohort. The median age was 60.0 years (IQR, 56.0–66.0), and 80.0% were male. No statistically significant between-group differences were observed in age, sex, smoking history, comorbidities, resected lobe, operation time, or histopathological subtype. Baseline demographic, surgical, and pathological characteristics are presented in Table 1.

Chest tube removal occurred earlier in the VATS cohort than in the open thoracotomy cohort: median (IQR), 4 (3–4) vs. 5 (4–5) days; *p* < 0.001.

### 3.2. Serum Cytokine Response

**Figure 1:**
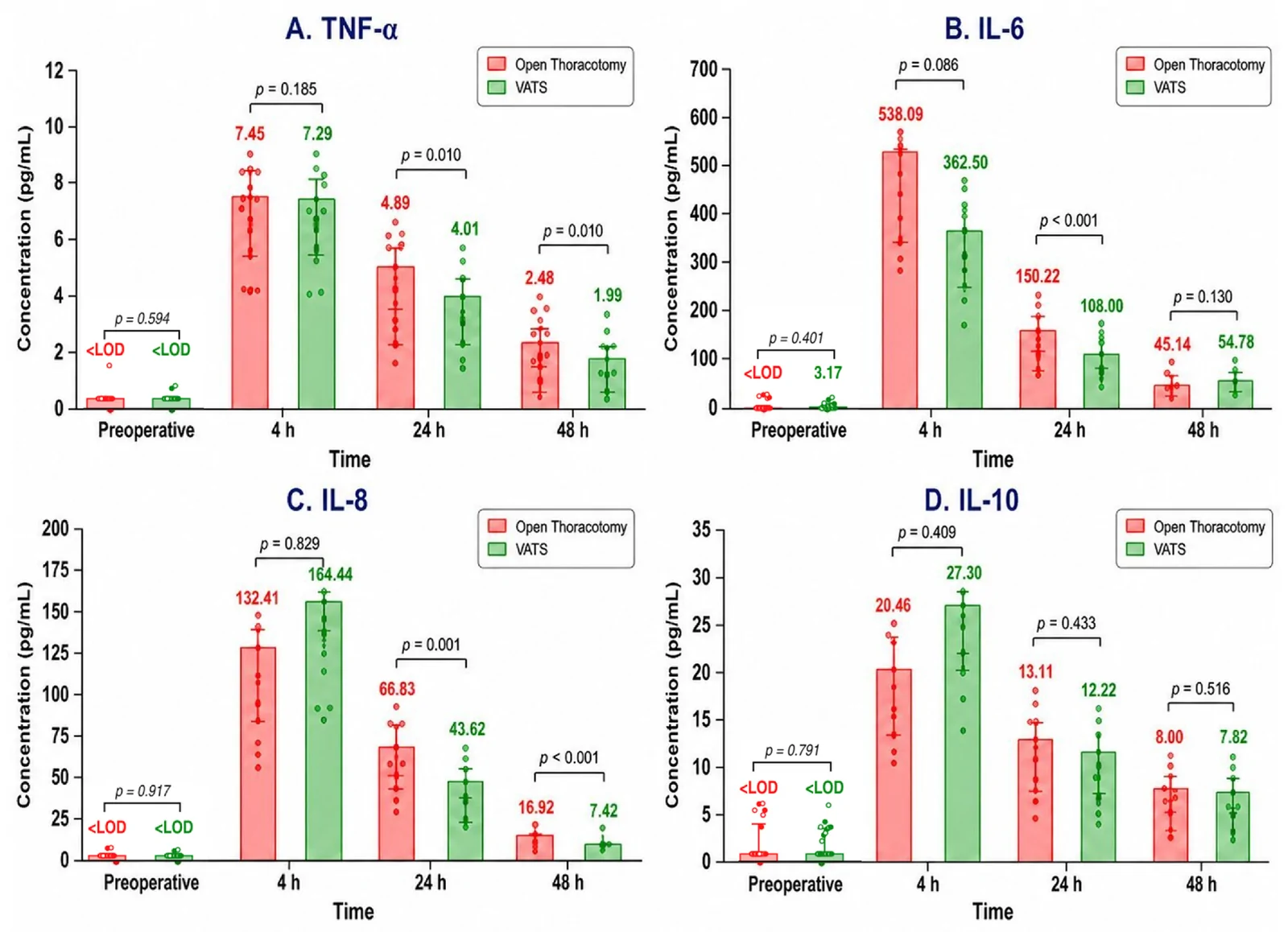
Serum cytokine profiles according to surgical approach. Red and green bars represent the open thoracotomy and video-assisted thoracoscopic surgery (VATS) groups, respectively. Bars show median values, error bars indicate interquartile ranges, and dots represent individual patient measurements. Values below the assay-specific limit of detection (LOD) were replaced with LOD/2 for statistical analysis and plotting, whereas values at or above the LOD were analyzed and plotted as measured. Medians below the assay-specific LOD are displayed as <LOD. Between-group comparisons were performed using the Mann–Whitney U test at each time point. TNF-α, tumor necrosis factor alpha; IL, interleukin.

Serum TNF-α, IL-6, IL-8, and IL-10 levels showed significant time-dependent changes in both groups (Friedman test; *p* < 0.001 for all cytokines), reaching peak values at postoperative 4 h and gradually decreasing at 24 and 48 h. No significant between-group difference was found at baseline or at postoperative 4 h. At postoperative 24 h, serum TNF-α (4.89 [IQR, 4.43–5.92] vs. 4.01 [IQR, 3.75–5.12] pg/mL, *p* = 0.010), IL-6 (150.22 [IQR, 136.90–194.66] vs. 108.00 [IQR, 100.46–125.88] pg/mL, *p* < 0.001), and IL-8 (66.83 [IQR, 57.70–82.82] vs. 43.62 [IQR, 32.89–50.59] pg/mL, *p* = 0.001) were significantly higher in the open thoracotomy group. At 48 h, differences persisted for TNF-α (2.48 [IQR, 1.86–3.04] vs. 1.99 [IQR, 1.19–2.23] pg/mL, *p* = 0.010) and IL-8 (16.92 [IQR, 15.09–23.99] vs. 7.42 [IQR, 6.44–11.30] pg/mL, *p* < 0.001). Serum IL-10 did not differ between groups at any time point (Figure 1). Longitudinal GEE analysis demonstrated significant approach-by-time interactions for serum TNF-α (Wald χ^2^ = 29.25, df = 3, *p* < 0.001), IL-6 (Wald χ^2^ = 12.91, df = 3, *p* = 0.005), and IL-8 (Wald χ^2^ = 30.66, df = 3, *p* < 0.001), whereas no significant interaction was observed for IL-10 (Wald χ^2^ = 0.85, df = 3, *p* = 0.838). These findings were materially unchanged after adjustment for age, smoking history, cardiac comorbidity, and pulmonary comorbidity (approach-by-time interaction: TNF-α, *p* < 0.001; IL-6, *p* < 0.001; IL-8, *p* < 0.001; IL-10, *p* = 0.889).

### 3.3. Pleural-Fluid Cytokine Response

**Figure 2:**
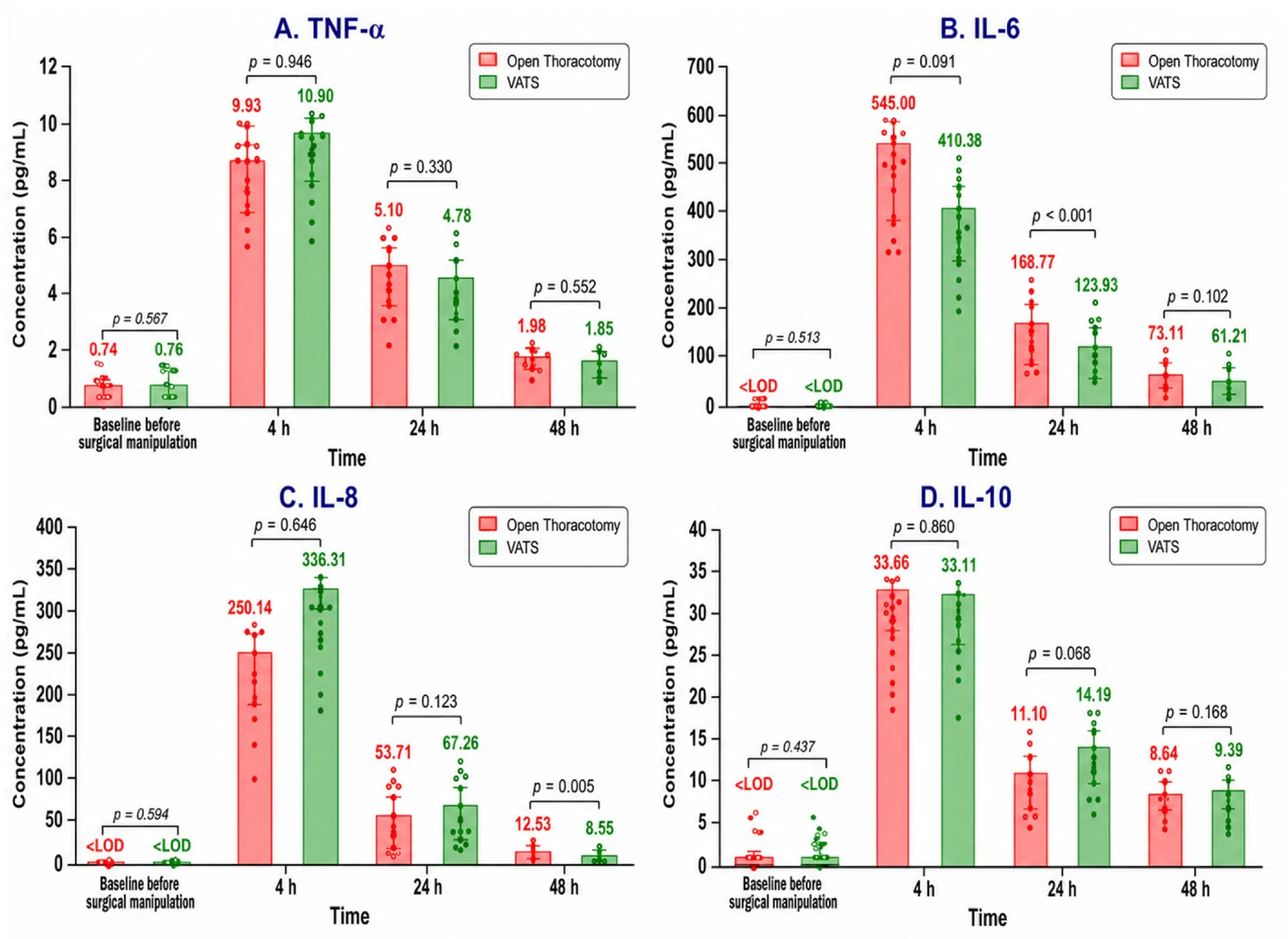
Pleural-fluid cytokine profiles according to surgical approach. Red and green bars represent the open thoracotomy and video-assisted thoracoscopic surgery (VATS) groups, respectively. Bars show median values, error bars indicate interquartile ranges, and dots represent individual patient measurements. Values below the assay-specific limit of detection (LOD) were replaced with LOD/2 for statistical analysis and plotting, whereas values at or above the LOD were analyzed and plotted as measured. Medians below the assay-specific LOD are displayed as <LOD. Between-group comparisons were performed using the Mann–Whitney U test at each time point. TNF-α, tumor necrosis factor alpha; IL, interleukin.

Pleural fluid TNF-α, IL-6, IL-8, and IL-10 also showed significant time-dependent changes in both groups (Friedman test; *p* < 0.001 for all cytokines), with peak values at postoperative 4 h and subsequent decreases. At baseline, several pleural-fluid cytokine measurements were below the assay-specific LOD, limiting the discriminatory value of between-group comparisons at this time point. No significant between-group difference was observed at postoperative 4 h. Pleural fluid IL-6 was higher in the open thoracotomy group at 24 h (168.77 [IQR, 160.30–174.70] vs. 123.93 [IQR, 119.02–134.17] pg/mL, *p* < 0.001), and pleural fluid IL-8 was higher at 48 h (12.53 [IQR, 9.72–13.43] vs. 8.55 [IQR, 6.07–9.82] pg/mL, *p* = 0.005). Other pleural fluid cytokines did not differ significantly between groups (Figure 2). Longitudinal GEE analysis showed a significant approach-by-time interaction for pleural-fluid IL-8 (Wald χ^2^ = 16.81, df = 3, *p* < 0.001), whereas no significant interactions were observed for TNF-α (Wald χ^2^ = 1.22, df = 3, *p* = 0.749) or IL-6 (Wald χ^2^ = 3.08, df = 3, *p* = 0.379). For IL-10, the approach-by-time interaction yielded *p* = 0.050 (Wald χ^2^ = 7.82, df = 3). After adjustment, the approach-by-time interaction remained significant for IL-8 (*p* < 0.001) and remained non-significant for TNF-α (*p* = 0.771) and IL-6 (*p* = 0.536); the IL-10 interaction was nominally significant (*p* = 0.023). This sensitivity-analysis finding was considered exploratory and should be interpreted cautiously. Thus, the between-group difference in pleural-fluid IL-6 at 24 h was confined to the pointwise comparison and was not accompanied by a significant approach-by-time interaction.

### 3.4. Bronchoalveolar-Lavage Cytokine Response

**Figure 3:**
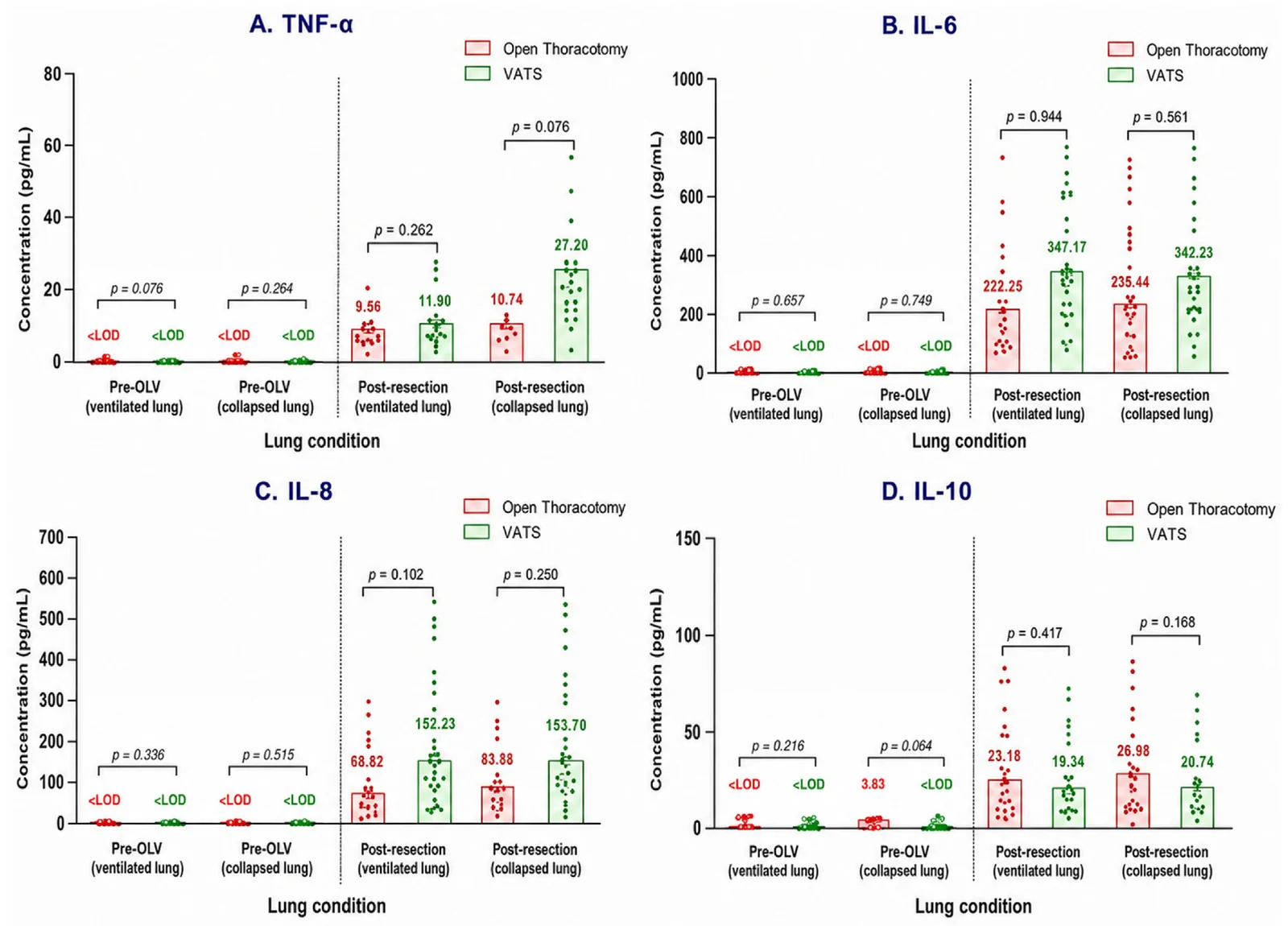
Bronchoalveolar-lavage cytokine profiles according to surgical approach and lung condition. Red and green bars represent the open thoracotomy and video-assisted thoracoscopic surgery (VATS) groups, respectively. Bars show median values, error bars indicate interquartile ranges, and dots represent individual patient measurements. Values below the assay-specific limit of detection (LOD) were replaced with LOD/2 for statistical analysis and plotting, whereas values at or above the LOD were analyzed and plotted as measured. Medians below the assay-specific LOD are displayed as <LOD. Surgical groups were compared within each sampling condition using the Mann–Whitney U test. The terms ventilated lung and collapsed lung refer to the ventilation status of the respective lungs during one-lung ventilation (OLV). BAL, bronchoalveolar lavage; TNF-α, tumor necrosis factor alpha; IL, interleukin.

At baseline, several BAL cytokine measurements were below the assay-specific LOD, limiting the discriminatory value of between-group comparisons at this time point. No significant between-group differences were observed in the post-resection BAL samples (Figure 3). Given the absence of adjustment for multiple comparisons, all pointwise between-group comparisons should be interpreted as exploratory.

## 4. Discussion

In this prospective multicompartment study, systemic, pleural, and alveolar cytokine responses after VATS and open thoracotomy were compared in patients undergoing lobectomy for NSCLC. In the present study, VATS was performed using a uniportal approach without rib spreading; therefore, the findings should be interpreted in the context of uniportal VATS. The measured baseline clinical and surgical characteristics were generally similar between the groups, allowing comparison of postoperative cytokine patterns; however, the non-randomized design does not permit definitive causal attribution of the observed differences to the surgical approach. Open thoracotomy was associated with a more pronounced systemic proinflammatory response than VATS, whereas differences in the pleural compartment were less consistent and no approach-related difference was observed at the BAL level. These findings suggest that the inflammatory response to surgical approach may differ across biological compartments.

Serum cytokines are widely used indicators of the systemic inflammatory response to surgical trauma. After lung resections, this response reflects tissue injury, one-lung ventilation, and perioperative immune activation [4,14,15,16]. In our study, TNF-α, IL-6, IL-8, and IL-10 peaked at postoperative 4 h and then decreased in both groups, indicating a similar early systemic response. However, the higher proinflammatory cytokine levels observed at later postoperative time points after open thoracotomy were accompanied by significant approach-by-time interactions for TNF-α, IL-6, and IL-8, indicating distinct temporal profiles between the surgical approaches. The roles of IL-6 in the acute-phase response, IL-8 in neutrophil chemotaxis, and TNF-α in early proinflammatory activation support the biological relevance of these findings [14,15,16]. Our results are consistent with studies reporting a more limited systemic cytokine response after VATS than after open thoracotomy [4,17]. Unlike the proinflammatory cytokines, serum IL-10 did not differ significantly between surgical approaches. This may reflect the distinct regulatory role and temporal kinetics of IL-10, whose postoperative response may not necessarily parallel those of IL-6, IL-8, and TNF-α. Previous studies have also reported heterogeneous IL-10 responses after VATS and thoracotomy [17,18].

Pleural fluid cytokine response reflects local inflammatory activation near the surgical field. Previous studies reported increased cytokine and chemokine levels in pleural fluid after lung resection and one-lung ventilation, indicating that this compartment may complement serum measurements [2,19]. In our study, pleural fluid TNF-α, IL-6, IL-8, and IL-10 peaked at postoperative 4 h and then decreased, demonstrating early and transient pleural inflammation. At individual sampling points, pleural IL-6 was higher after open thoracotomy at 24 h and IL-8 at 48 h. In the longitudinal analysis, however, an approach-by-time interaction was observed for IL-8 but not for IL-6, indicating that temporal divergence in the pleural compartment was more evident for IL-8. One possible explanation is the greater extent of chest-wall and pleural tissue manipulation associated with open thoracotomy. This agrees with studies indicating that VATS may induce a more limited inflammatory response through less mechanical trauma [4,20]. However, the lack of consistent pointwise differences in other pleural cytokines suggests that pleural inflammation may also be affected by resection extent, tumor biology, one-lung ventilation, drainage dynamics, and individual immune response [2,19].

BAL findings indicate that local pulmonary inflammation can be evaluated independently of systemic response. During one-lung ventilation, the ventilated lung may be affected by mechanical stress, hyperperfusion, and oxidative stress, while the collapsed lung may be influenced by surgical manipulation, hypoxia, re-expansion, and ischemia-reperfusion processes. Thus, BAL cytokine response reflects not only incision size but also lung-specific pathophysiological effects in ventilated and collapsed lungs during OLV [2,21,22]. In our study, cytokines were measured separately in both lung conditions, but no significant between-group difference was observed in any BAL sampling condition. Taken together, these findings did not demonstrate a statistically significant difference in early alveolar cytokine profiles between the two surgical approaches during the immediate perioperative period; later alveolar responses could not be assessed because BAL sampling was not extended beyond this period. Jones et al. [17] similarly reported lower serum cytokine responses after VATS than after thoracotomy, with no clear difference between approaches in BAL samples.

From a practical perspective, the lower systemic cytokine responses and the more limited differences observed in the pleural compartment after VATS are consistent with the reduced surgical-access trauma associated with minimally invasive approaches [4,20]. The absence of an approach-related difference in BAL cytokines is also consistent with the continued importance of lung-protective one-lung ventilation during both VATS and open thoracotomy [2,21,22]. These findings do not support routine perioperative cytokine monitoring or selection of the surgical approach based on cytokine levels alone; rather, they identify serum and pleural fluid as potentially informative compartments for future studies linking surgical inflammation with postoperative recovery and complications.

This study has several limitations. First, the non-randomized design and patient-preference-based selection may have introduced selection bias and residual confounding. However, only patients considered technically suitable for either surgical approach were enrolled, and the eligibility criteria, oncological surgical principles, sampling procedures, and perioperative protocols were standardized between the groups. Consecutive eligible patients were enrolled within each surgical cohort until the planned sample size was reached. Although the prospective design and standardized sampling protocol strengthen the reliability of the study, the single-center design and limited sample size require validation of the findings in larger series. Given the limited sample size, the secondary pleural-fluid and BAL analyses may have been underpowered to detect smaller between-group differences. Accordingly, the absence of significant differences in BAL cytokine levels should not be interpreted as evidence of equivalence. Sex-stratified analyses were not performed because only eight women were enrolled, precluding reliable subgroup estimates. The restriction of cytokine measurements to the early perioperative period did not allow evaluation of late inflammatory changes. Although sensitivity analyses adjusted for selected baseline clinical characteristics, residual confounding from unmeasured or incompletely characterized factors cannot be excluded. In addition, potentially relevant perioperative variables such as body mass index, preoperative pulmonary function, intraoperative blood loss, and duration of one-lung ventilation were not included in the present analysis and may have influenced cytokine kinetics. The numerous compartment-, analyte-, and time-specific cytokine analyses were not adjusted for multiplicity; therefore, the reported *p*-values should be considered nominal, and the findings interpreted as exploratory. Nevertheless, simultaneous analysis of serum, pleural fluid, and BAL samples enabled separate assessment of surgical approach-related inflammatory responses at systemic, local, and alveolar levels. In this respect, the study demonstrates that the inflammatory response after VATS and open thoracotomy should be interpreted not only through systemic circulation but also by considering different compartments.

## 5. Conclusions

This prospective multicompartment study suggests that open thoracotomy was associated with a more pronounced postoperative systemic proinflammatory response than VATS. Differences in the pleural compartment were more limited, with a longitudinal difference observed for IL-8, whereas the IL-6 difference was confined to the 24-h pointwise comparison. Conversely, no significant between-group differences were detected in early BAL cytokine profiles at the sampled time points. These results indicate that assessing inflammatory responses separately across different biological compartments may provide deeper insight into how surgical trauma differs between minimally invasive and open lobectomy.

## Figures and Tables

**Table 1:** Demographic, clinical, surgical, and pathological characteristics according to surgical approach.

Variable	Thoracotomy (*n* = 20)	VATS (*n* = 20)	Total (*n* = 40)	*p*-Value
Age, years, median (IQR)	60.0 (54.0–65.5)	60.5 (59.0–66.0)	60.0 (56.0–66.0)	0.407 †
Sex, *n* (%)				1.000 ‡
Male	16 (80.0%)	16 (80.0%)	32 (80.0%)	
Female	4 (20.0%)	4 (20.0%)	8 (20.0%)	
Smoking history, *n* (%)				0.451 ‡
Ever smoker, current/former	17 (85.0%)	14 (70.0%)	31 (77.5%)	
Never smoker	3 (15.0%)	6 (30.0%)	9 (22.5%)	
Cardiac comorbidity, *n* (%)	9 (45.0%)	14 (70.0%)	23 (57.5%)	0.110 ‡
Pulmonary comorbidity, *n* (%)	9 (45.0%)	8 (40.0%)	17 (42.5%)	0.749 ‡
Diabetes mellitus, *n* (%)	7 (35.0%)	6 (30.0%)	13 (32.5%)	0.736 ‡
Resected lobe, *n* (%)				0.815 ‡
Left lower lobe	7 (35.0%)	8 (40.0%)	15 (37.5%)	
Left upper lobe	4 (20.0%)	4 (20.0%)	8 (20.0%)	
Right upper lobe	5 (25.0%)	3 (15.0%)	8 (20.0%)	
Right lower lobe	4 (20.0%)	4 (20.0%)	8 (20.0%)	
Right middle lobe	0 (0.0%)	1 (5.0%)	1 (2.5%)	
Chest tube removal time, days, median (IQR)	5 (4–5)	4 (3–4)	4 (4–5)	<0.001 †
Operation time, min, median (IQR)	190 (172.5–200)	180 (150–200)	180 (150–200)	0.289 †
Histology, *n* (%)				0.766 ‡
Adenocarcinoma	10 (50.0%)	11 (55.0%)	21 (52.5%)	
Squamous cell carcinoma	8 (40.0%)	6 (30.0%)	14 (35.0%)	
Large-cell carcinoma	2 (10.0%)	3 (15.0%)	5 (12.5%)	
Clinical stage, *n* (%)				0.525 ‡
Stage I	12 (60.0%)	10 (50.0%)	22 (55.0%)	
Stage II	8 (40.0%)	10 (50.0%)	18 (45.0%)	
Pathological stage, *n* (%)				0.342 ‡
Stage I	11 (55.0%)	8 (40.0%)	19 (47.5%)	
Stage II	9 (45.0%)	12 (60.0%)	21 (52.5%)	
Pathological nodal status, *n* (%)				0.661 ‡
pN0	16 (80.0%)	18 (90.0%)	34 (85.0%)	
pN1	4 (20.0%)	2 (10.0%)	6 (15.0%)	
pN2	0 (0.0%)	0 (0.0%)	0 (0.0%)	

Values are presented as median (interquartile range [IQR]) or *n* (%), as appropriate. † Mann–Whitney U test. ‡ Pearson chi-square or Fisher’s exact test, as appropriate. A *p*-value < 0.05 was considered statistically significant. VATS, video-assisted thoracoscopic surgery.

## Data Availability

The data presented in this study are available from the corresponding author on reasonable request. The data are not publicly available because they contain potentially identifiable clinical information and are subject to institutional and ethical restrictions.
